# Statistical and sequence learning lead to persistent memory in children after a one-year offline period

**DOI:** 10.1038/s41598-021-90560-5

**Published:** 2021-06-14

**Authors:** Eszter Tóth-Fáber, Karolina Janacsek, Dezső Németh

**Affiliations:** 1 Doctoral School of Psychology, ELTE Eötvös Loránd University, Izabella utca 46, 1064 Budapest, Hungary; 2 Institute of Psychology, ELTE Eötvös Loránd University, Izabella utca 46, 1064 Budapest, Hungary; 3 Brain, Memory and Language Research Group, Institute of Cognitive Neuroscience and Psychology, Research Centre for Natural Sciences, Magyar tudósok körútja 2, 1117 Budapest, Hungary; 4 Centre for Thinking and Learning, Institute for Lifecourse Development, School of Human Sciences, Faculty of Education, Health and Human Sciences, University of Greenwich, Old Royal Naval College, Park Row, 150 Dreadnought, London, SE10 9LS UK; 5 Lyon Neuroscience Research Center (CRNL), INSERM U1028, CNRS UMR5292, Centre Hospitalier Le Vinatier, Université de Lyon 1, Université de Lyon, Bâtiment 462 - Neurocampus 95 boulevard Pinel, 69675 Bron, Lyon, France

**Keywords:** Human behaviour, Consolidation, Cognitive neuroscience

## Abstract

Extraction of environmental patterns underlies human learning throughout the lifespan and plays a crucial role not only in cognitive but also perceptual, motor, and social skills. At least two types of regularities contribute to acquiring skills: (1) statistical, probability-based regularities, and (2) serial order-based regularities. Memory performance of probability-based and/or serial order-based regularities over short periods (from minutes to weeks) has been widely investigated across the lifespan. However, long-term (months or year-long) memory performance of such knowledge has received relatively less attention and has not been assessed in children yet. Here, we aimed to test the long-term memory performance of probability-based and serial order-based regularities over a 1-year offline period in neurotypical children between the age of 9 and 15. Participants performed a visuomotor four-choice reaction time task designed to measure the acquisition of probability-based and serial order-based regularities simultaneously. Short-term consolidation effects were controlled by retesting their performance after a 5-h delay. They were then retested on the same task 1 year later without any practice between the sessions. Participants successfully acquired both probability-based and serial order-based regularities and retained both types of knowledge over the 1-year period. The successful retention was independent of age. Our study demonstrates that the representation of probability-based and serial order-based regularities remains stable over a long period of time. These findings offer indirect evidence for the developmental invariance model of skill consolidation.

## Introduction

Detecting and extracting various kinds of regularities embedded in our environment is a fundamental component underlying human learning in all ages, enabling us to adapt to our surroundings and to predict future events^1–4^. Extraction of regularities is argued to be the basis of several motor and cognitive skills, including language^3,5–9^. The initially unstable representations of the detected and extracted regularities are converted into a more stable form via consolidation, allowing information to be preserved and retained later^10^. Several studies investigated the consolidation of regularities and skills with a 1-min, 1-h, 4-h, 12-h, 24-h or 1-week delay^11–19^. Although everyday experiences suggest that the representation of the acquired regularities and skills is persistent even for a more extended period (months or years), it has been rarely tested empirically, especially from a developmental perspective. In the present study, we aim to investigate the long-term (1-year) consolidation of two types of regularities in neurotypical children.

Behaviorally, consolidation is measured by contrasting memory performance at the end of the learning session with performance at the beginning of a subsequent testing session, without additional practice between the two sessions (i.e., during the offline periods). Consolidation can be expressed by successfully retained knowledge after the offline period (no forgetting, i.e., performance is similar in the learning and testing sessions) or by learning-dependent, delayed performance gains after the offline period, termed offline learning (i.e., performance is better in the testing session than in the learning session)^20^. The present study follows this well-established behavioral test protocol to assess long-term memory performance and implements a 1-year offline period between the sessions.

The available information in our environment, which can be detected, extracted, and consolidated is diverse; thus, our brain has to process several information streams simultaneously during both learning and consolidation. Learning of regularities is not a monolithic process. Previous empirical studies suggested that the acquisition of at least two types of regularities can be differentiated: (1) statistical, probability-based regularities, and (2) serial order-based regularities^17,21–23^. Therefore, it has been proposed that humans organize the regularities embedded in the environment in separate hypothesis spaces^3,24^; one such hypothesis space is based on probabilities, while another is based on deterministic rules (i.e., serial order-based regularities). While on the level of transitional probabilities, these learning processes may seem highly similar, where the former can be viewed as the acquisition of transitional probabilities that are less than one and the latter as the acquisition of transitional probabilities that equal one, differences between them have been shown both on the behavioral and neural levels, providing support for their distinction^17,21,22^. Probability-based regularities are picked up rapidly, while learning of serial order-based information follows a more gradual trajectory^17,21^; they also manifest differently on the level of event-related potentials^22,23^ and show different neural oscillations during consolidation^17,25^.

The consolidation of probability-based and/or serial order-based regularities has been studied previously. Retained knowledge has been found after 1-h, 12-h, 24-h, or even 1-week offline period in healthy adults^13–17,19^. Long-term consolidation has received less attention, with only a few studies investigating the effect of month- or year-long offline periods: Romano, et al. ^26^ and Kóbor, et al. ^27^ both showed persistent representation of regularities after a 1-year offline period in healthy adults. However, both studies employed a task^26,27^, which, although measures both probability-based and serial order-based information, is not well-suited to dissect these regularities in the same time window. To the best of our knowledge, only two studies investigated the consolidation differences between probability-based and serial order-based regularities, but they administered a 1-h offline period only^17,25^. Both Simor et al.^17^ and Zavecz et al.^25^ found retained statistical and serial-order knowledge after the offline period. Altogether, prior studies typically incorporated only short-term (from minutes to week) offline periods in their design; therefore, the long-term consolidation of probability-based and serial order-based information is not well understood yet. Here, we aimed to fill this gap by investigating the simultaneous consolidation of these regularities over a 1-year period.

The consolidation of probability-based or serial order-based information is even less understood in children. An ideal avenue to achieve a deeper understanding of cognitive processes and functions is to examine them from a developmental perspective^28^. Studies on typical and atypical development can pave the way towards grasping underlying processes of learning and memory consolidation. Learning of probability-based regularities might be age-variant with better performance in children up to the age of 12^21,29,30^, whereas learning of serial order-based regularities might be comparable in children and adults^21^. Most of the studies examining the consolidation of these regularities in children either focused on solely probability-based^31^ or solely serial order-based regularities^32,33^ or used paradigms that intermix them^34–37^. Retained information (i.e., no forgetting) has been found in neurotypical children following 11-h^31^, 16-h^35,37^, 24-h^32^ and 3-day^34^ offline periods. Hedenius et al.^36^ showed offline learning after a 24-h delay and Desmottes et al.^33^ found offline learning following 24-h and 1-week offline periods. To the best of our knowledge, the long-term (1-year) consolidation of probability-based or serial order-based information has not yet been investigated in children. Age-variant learning of probability-based regularities and successful 1-year retention in healthy adults^26,27^ raises the question of whether long-term consolidation is successful in children as well.

To sum up, in child population, the long-term (1-year) consolidation of probability-based and serial order-based information has not been assessed yet. In the present study, we used the cued version of the Alternating Serial Reaction Time task^21,38^ which enables us to simultaneously measure these two regularities. In this framework, statistical learning refers to the acquisition of short-range, temporally distributed probability-based information between visual stimuli. Sequence learning refers to the acquisition of serial order-based information, where participants are exposed to stimuli that repeatedly occur in the same deterministic order, incorporated with random stimuli (hence, creating an alternating sequence structure). Our study aims to examine 1-year consolidation of probability-based and serial order-based regularities in children between the age of 9 and 15 with a task designed to measure the acquisition of these regularities simultaneously. This particular age range was chosen in order to examine consolidation both in childhood and adolescence, hence, participants from pre-adolescence, early- and mid-adolescence were included. Based on the previous studies, we expect successful retention of both probability-based and serial order-based information following a 1-year offline period.

## Methods

### Participants

Seventy-eight children between the age of 9 and 15 participated in our study from local schools. Three participant had missing data on the ASRT task due to technical difficulties; three children’s caregiver reported psychiatric condition; one child did not have corrected-to-normal vision during one session of the assessments; and one children showed extremely low average accuracy according to Tukey^39^ criterion (more than 3 times the interquartile range from the quartiles) consistently throughout the ASRT task. These eight participants were excluded from the analyses. The final sample consisted of 70 participants (M_age_ = 11.99 years, SD_age_ = 1.61 years; 37 boys, 33 girls).

Participants performed in the normal range on standard neuropsychological tests (Wisconsin Card Sorting Task^40,41^ [WCST, percentage of perseverative errors]: *M* = 14.12%, *SD* = 5.90%; Counting Span task^42,43^: *M* = 3.17, *SD* = 0.78). Due to technical problems, data of three participants on the WCST and data of one participant on the WCST and Counting Span task is missing. Handedness was measured by the Edinburgh Handedness Inventory^44^ (EHI). Due to a technical error, EHI of one participant is missing. The Laterality Quotient (LQ) of the sample varied between -100 and 100 (where -100 means compete left-handedness and 100 means complete right-handedness) with a mean of 76.21 (*SD* = 36.50).

Furthermore, caregivers of participants completed a parental questionnaire regarding socioeconomic status (SES) and health-related questions (i.e., whether the child has any neurological, psychiatric, or neurodevelopmental disorder). Caregivers of one participant did not provide information about their socioeconomic status (SES), therefore, data of one participant is missing. SES was determined by how many years the caregivers spent in formal education. We calculated the caregivers’ average formal education based on both parents’ education. In case of five participants, we only had information about one caregiver. The range of formal education of caregivers was between 9.5 and 27 years, with a mean of 16.61 years (*SD* = 3.85 years). Caregivers were also asked to fill in the Strength and Difficulties Questionnaire^45^ (SDQ) which measures hyperactivity, conduct problems, emotional problems, and difficulties in peer relationships. SDQ of six participants is missing. Total problem score measured in our sample was 7.94 (*SD* = 5.38), which is well in the normal range of typically developing children^46^. All participants in the final sample had normal or corrected-to-normal vision, and none of the children had any neurological, psychiatric, or neurodevelopmental disorders according to parental reports.

Caregivers of all participants provided informed written consent, and children provided informed verbal consent to participate in the study before enrollment. The study was approved by the research ethics committee of Eötvös Loránd University, Budapest, Hungary (2018/239), and was conducted in accordance with the Declaration of Helsinki.

### Task

**Figure 1:**
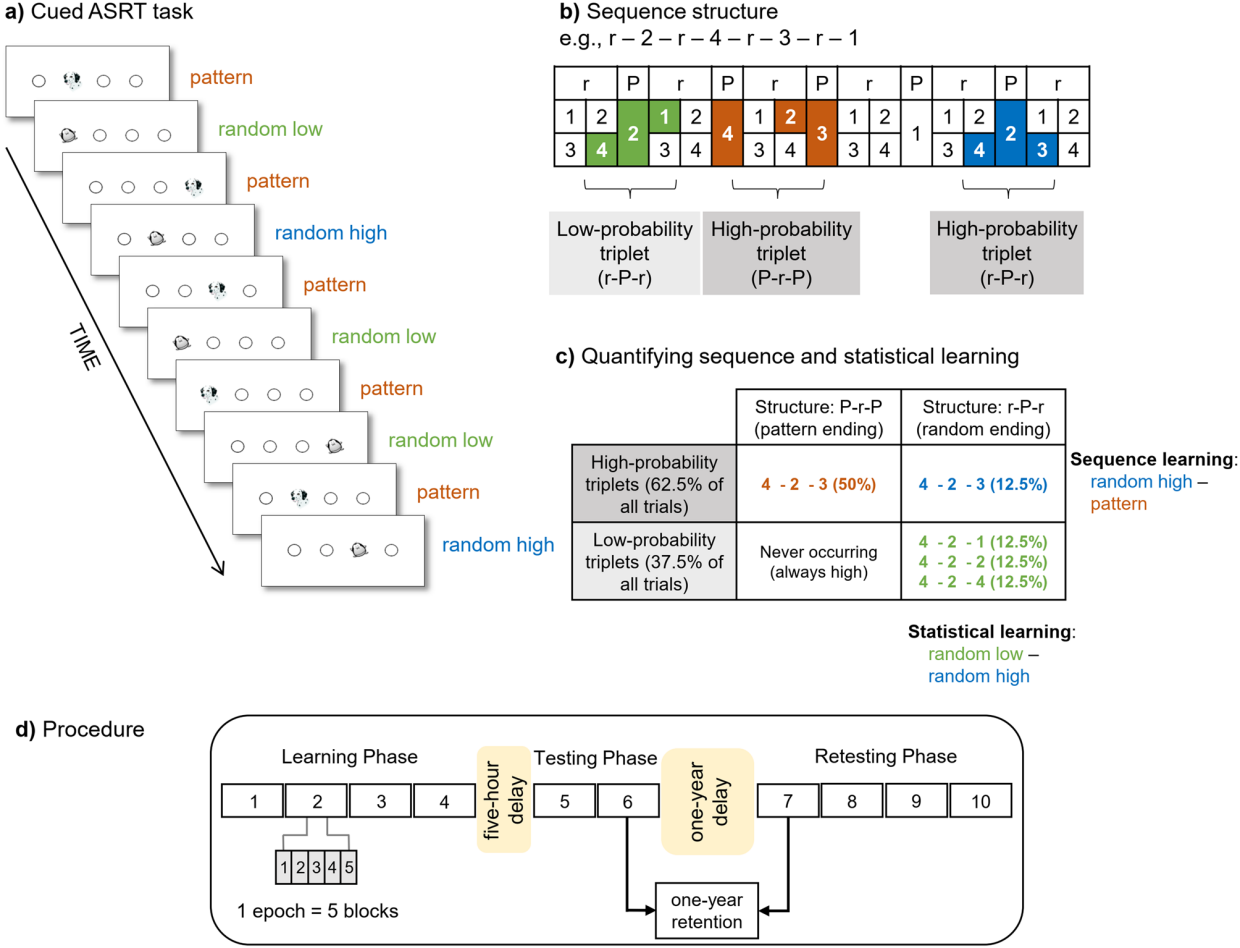
The cued Alternating Serial Reaction Time (ASRT) task and experimental procedure. (**a**) Pattern and random trials were presented in an alternating fashion. Pattern trials were marked by a picture of a dog’s head, and random trials were marked by a picture of a penguin. (**b**) An example of the sequence structure. Numbers indicate pattern trials, and ‘r’ indicates a randomly selected location out of the four possible ones. The alternating sequence makes some runs of three consecutive trials (labeled as triplets) more probable than others, called high-probability and low-probability triplets, respectively. Among high-probability triplets, the last element of the triplet can be either pattern or random. Based on this, we could determine pattern triplets that are always of high probability (orange shading in panel B and orange font in panel C) and random high-probability triplets (blue shading in panel B and blue font in panel C). Among low-probability triplets, only random low-probability triplets can occur (green shading in panel B and green font in panel C). (**c**) The underlying learning processes measured by the task. Statistical learning is calculated by contrasting the accuracies or RTs on the random high and random low trials (blue vs. green, the right column of the table). Sequence learning is quantified by contrasting the accuracies or RTs on the pattern and random high trials (orange vs. blue, the top row of the table). The table presents the calculation of learning processes on RT data. (**d**) The design of the experiment. The experiment was composed of three sessions. The Learning Phase consisted of four epochs (one epoch contained 5 blocks, and each block consisted of 85 stimuli), followed by a 5-h offline period then the two-epoch-long Testing Phase on the same day. The Retesting Phase with four epochs was administered ca. one year later. Figure 1A, 1B, and 1C are adapted from Nemeth, et al. 21, Fig. 1D is adapted from Kóbor, et al. 27.

The detection and extraction of probability-based and serial order-based regularities was measured by the cued version of the Alternating Serial Reaction Time (ASRT) task^21,38^. In this task, four equally spaced, horizontally arranged empty circles were presented on the screen, and a stimulus (either a dog’s head or a penguin) appeared in one of the possible locations (i.e., in the empty circles) (Fig. 1a). The task was bimanual and participants were asked to press the corresponding key as accurately and as fast as they could using the index and middle fingers of both hands. After the response of the participant, the next target appeared 120 ms later.

The presentation of the stimuli followed an eight-element alternating sequence within which pattern and random elements alternated with each other (e.g., 1-r-2-r-4-r-3-r, where numbers indicate the locations from left to right and ‘r’ indicates a randomly selected location out of the four possible ones). In the cued ASRT task, pattern and random elements are marked by different visual stimuli, where pattern elements are denoted by the dog’s head, and random elements are indicated by the penguins. Participants were informed about the *presence* of the sequence and about the fact that the appearance of dogs always follows a predetermined pattern, while penguins always appear in random order. They were *not* informed about the exact sequence; they were instructed to find the pattern of the dogs’ appearance to improve their performance. The alternating sequence makes six different sequence variations: 1-r-2-r-3-r-4-r, 1-r-2-r-4-r-3-r, 1-r-3-r-2-r-4-r, 1-r-3-r-4-r-2-r, 1-r-4-r-2-r-3-r, and 1-r-4-r-3-r-2-r. These permutations can start at any location (e.g., 1-r-2-r-3-r-4-r and 2-r-3-r-4-r-1-r are identical sequence permutations). One of the permutations was selected for each participant in a counterbalanced fashion across participants. For a given participant, the permutation remained the same through all sessions. The stimuli were presented in blocks, each block consisting of 85 trials. Each block started with five random trials for practice, then one of the eight-element alternating sequence was presented ten times.

In the task, three successive trials are referred to as triplets. Due to the alternating sequence in the task, some triplets are more probable than others. Each trial is categorized as the last element of a triplet in a moving window manner, which means that a given trial is characterized as the first element of a given triplet, as the second element of the following triplet and also as the last element of the next triplet, irrespective of whether it is a pattern or random trial. In the example sequence of Fig. 1b, 2-r-4-r-3-r-1-r (numbers indicate the locations from left to right and ‘r’ indicates a randomly selected location out of the four possible ones), 2-X-4, 4-X-3, 3-X-1 and 1-X-2 (where X represents the middle element of the triplet) appeared with a higher probability because their third element could have been either pattern or random. Note that here, we use X to indicate the middle element of the triplet because for example 4-X-3 can appear both as 4-2-3 (Pattern–random–Pattern) where the first and last elements are part of the predetermined pattern and as 4-2-3 (random–Pattern–random) where the first and last elements are random, and the middle element is part of the predetermined pattern (see also Fig. 1b,c). In contrast, triplets such as 4-2-1 or 4-2-2 occurred with a lower probability because their third element could have been only random (that is, random–Pattern–random structure). The former triplet types are called “high-probability” triplets, while the latter types are labeled as “low-probability” triplets^38^.

Besides probability, another important aspect of the elements is their structure, meaning whether they are pattern or random elements. High-probability triplets can be differentiated based on their last element being a pattern element or a random element. The third element of low-probability triplets can only be random since pattern elements always appear with high probability. Importantly, performance is not operationalized on the level of triplets; instead, performance (i.e., accuracy or reaction time) is always calculated only on the last element of a triplet. Each element (i.e., trial) was categorized as the third element of either a high-probability or a low-probability triplet and also either as pattern or random elements (note that they are also visually distinguishable).

There are 64 unique triplets in the task, including all Pattern–random–Pattern (50%) and random–Pattern–random (50%) triplets; 16 triplets are high-probability triplets, and 48 triplets are low-probability ones. Regarding high-probability triplets, there are four possible combinations in regard to the first and third elements of the triplet (for the example sequence: 2-r-4-r-3-r-1-r, 2-X-4, 4-X-3, 3-X-1 and 1-X-2) with four possible locations for the middle element. In detail, the high-probability triplet of 4-X-3 can be 4-1-3, 4-2-3, 4-3-3 and 4-4-3. Since high-probability triplets can occur as Pattern–random–Pattern (50%) and by 1/4 chance as random–Pattern–random (12.5%), these triplets constitute 62.5% of all trials (Fig. 1c). As for low-probability triplets, the first and the second element of the triplet can appear on any of the four locations, whereas the last element has three possible locations as the fourth one corresponds to a high-probability triplet. Thus, low-probability triplets constitute 37.5% of all trials. As noted above, all low-probability triplets have a random–Pattern–random structure. On the level of unique triplets, high-probability triplets are five times more probable than the low-probability ones (4% [62.5%/16] vs. 0.8% [37.5%/48]).

Altogether, three trial types can be distinguished: (1) trials that belong to the predetermined sequence and are the last element of a high-probability triplet labeled as *pattern trials* (such as 4-2-3 in Fig. 1b,c marked with orange); (2) random elements that are the last element of a high-probability triplet called *random high trials* (such as 4-2-3 in Fig. 1b,c marked with blue); and (3) random elements that are the last element of a low-probability triplet labeled as *random low trials* (such as 4-2-1 in Fig. 1b,c marked with green).

Prior studies have demonstrated that participants show gradually faster responses to high-probability triplets than low-probability ones as the task progresses (i.e., triplet learning in the original, uncued version of the ASRT task). However, as high-probability triplets consist of both pattern and random triplets, serial-order knowledge cannot be measured by comparing merely the high- and low-probability triplets. It is important to note that in the uncued ASRT task, the underlying structure is identical to the one in the cued ASRT task, however, pattern and random stimuli are not visually distinguishable^38^. Due to the identical underlying structure, the dissection of probability-based and serial-order based regularities is possible in the uncued ASRT task, but excessive training (i.e., 4-day-long training) is needed for the acquisition of serial-order knowledge^38,47^. The visual distinction of pattern and random trials ensures that the acquisition of both probability-based and serial-order based regularities—also referred to as statistical and sequence learning, respectively^21,38^—can be measured within the same time frame.

Statistical learning is quantified by the difference in accuracy or reaction times (RTs) between random high- and random low-probability trials. These trials share the same sequence properties as they are both random but differ in statistical properties as, on the level of unique triplets, the former ones are more probable than the latter ones (see details above). Sequence learning is measured by the difference in accuracy or RTs between pattern and random high-probability trials. The statistical properties of these trials are identical as they are both highly probable, while their sequence properties differ as pattern trials are part of the predetermined sequence. In conclusion, statistical learning refers to the acquisition of purely probability-based information, while sequence learning captures the acquisition of serial order-based information (Fig. 1c).

### Procedure

The experiment was composed of three sessions. The first two sessions were administered on the same day with a 5-h delay between them, while the third session was administered ca. one year later (*M*_delay_ = 53.08 weeks, *SD*_delay_ = 2.39 weeks, between 47.95 and 60.24 weeks, Fig. 1d). The ASRT task was administered in all three sessions. In the Learning Phase, participants completed 20 blocks, which, during the statistical analyses, were collapsed into epochs, each containing five blocks. The Testing Phase consisted of 10 blocks (i.e., two epochs), while the Retesting Phase contained 20 blocks (i.e., four epochs) (Fig. 1d). Participants were assessed in a quiet room in their school. During the 5-h offline period on the first day, they continued with their school activities such as classes and extracurricular activities. At the end of the first day (i.e., after the Learning and Testing Phases), participants were not informed that they would perform the task 1 year later.

### Statistical analyses

Statistical analyses were carried out by SPSS version 25.0 software (SPSS, IBM) and by JASP 0.11.1.0. software^48^. Based on previous studies using the ASRT task^17,21,22^, we firstly collapsed the blocks of the task into epochs, with each epoch consisting of five blocks. This way, the Learning Phase contained four epochs, the Testing Phase contained two epochs, while the Retesting Phase consisted of four epochs. Epochs are labeled consecutively (from 1 to 10, Fig. 1d). From the analysis, two types of low-probability triplets were excluded: repetitions (e.g., 111, 222) and trills (e.g., 121, 242), as participants often show pre-existing tendencies to them ^13,49^. As described above in the task description, each trial was determined as the last trial of a pattern, random high, or random low triplet. Mean accuracy (ratio of correct responses) and median RT (for correct responses) were calculated for each participant and each epoch, separately for the three types of trials (i.e., pattern, random high and random low trials). Based on the three trial types, statistical and sequence learning can be assessed by the cued ASRT task^21^ (for further details, see the task’s description above). Analyses and results concerning accuracy are presented in the Supplementary Material; here, we focus on RT data.

Prior developmental studies showed that age has a large effect on average RTs, with younger children showing slower RTs [e.g.,^29,30,50^]. To test this, we first calculated average RTs over the 10 epochs (i.e., RT data were calculated on all correct trials, irrespective of trial types). We then correlated the average RTs with age: the analysis revealed significant negative correlation (*r*(68) = −0.54, *p* < 0.001), showing that younger children were slower on the task. To control for the effect of average RT differences related to age on learning and consolidation of knowledge, we transformed the data in the following way. We divided each participants’ raw RT values of each trial type and each epoch by their own average performance (i.e., average RT) in the first epoch of the task [for a similar approach, see^51,52^]. Participants’ performance was around 1 at the beginning of the task and changed as the task progressed. Values above 1 meant that responses were slower on a given trial type than average RTs in the very first epoch of the task, while values below 1 indicated faster responses on a given trial type compared to average RTs in the first epoch. We conducted all analyses on standardized RT data.

Statistical learning score in the Learning Phase and memory scores in the Testing and Retesting Phases were quantified as the difference between random high and random low trial types in RT (RT for random low minus RT for random high trials). The learning and memory scores of sequence learning were calculated as the difference between pattern and random high trial types in RT (RT for random high minus RT for pattern trials; Fig. 1c). Higher scores indicate larger statistical or sequence learning/memory. To assess learning and the retention of knowledge, repeated-measures ANOVAs and paired-samples t-tests were conducted on standardized RT data, separately for statistical and sequence learning. The Greenhouse–Geisser epsilon (ε) correction was used when necessary. Original *df* values and corrected *p* values (if applicable) are reported with partial eta-squared (*η*^2^_*p*_) as a measure of effect size. For correlation analyses, in case of normal distribution, Pearson’s correlation was employed. When the assumption of normal distribution was violated, Spearman correlation was used for frequentist statistics and Kendall’s Tau-b correlation was used for Bayesian statistics.

In conjunction with the frequentist analyses, we performed Bayesian paired-samples t-tests and calculated the Bayes Factor (BF) for the relevant comparisons as well. The BF is an excellent tool that helps to conclude whether the collected data supports the null-hypothesis (H_0_) or the alternative hypothesis (H_1_)^53^. BFs can be particularly relevant in consolidation studies where memory retention is reflected by evidence supporting the H_0_ rather than H_1_^54^. In this case, H_0_ states the lack of difference between the mean of the memory scores before and after the offline period, while H_1_ states that the mean of the memory scores differ. Here, we report BF_01_ values, which were calculated using the JASP software [version 0.11.1.0.^48^]. According to Wagenmakers, et al.^53^ BF_01_ values between 1 and 3 indicate anecdotal evidence, values between 3 and 10 suggest substantial evidence and values larger than 10 indicate strong evidence for H_0_. Values between 1 and 1/3 suggest anecdotal evidence, values between 1/3 and 1/10 indicate substantial evidence, and values below 1/10 indicate strong evidence for H_1_. Values around 1 do not support either hypothesis.

## Results

### Prerequisite of memory consolidation

To assess memory consolidation, significant learning has to occur preceding the offline period. Therefore, as a first step, we conducted repeated-measures ANOVAs on the Learning Phase to confirm that significant learning has occurred concerning both statistical and sequence learning. ANOVAs were conducted on standardized RT data, separately for statistical and sequence learning.

**Figure 2:**
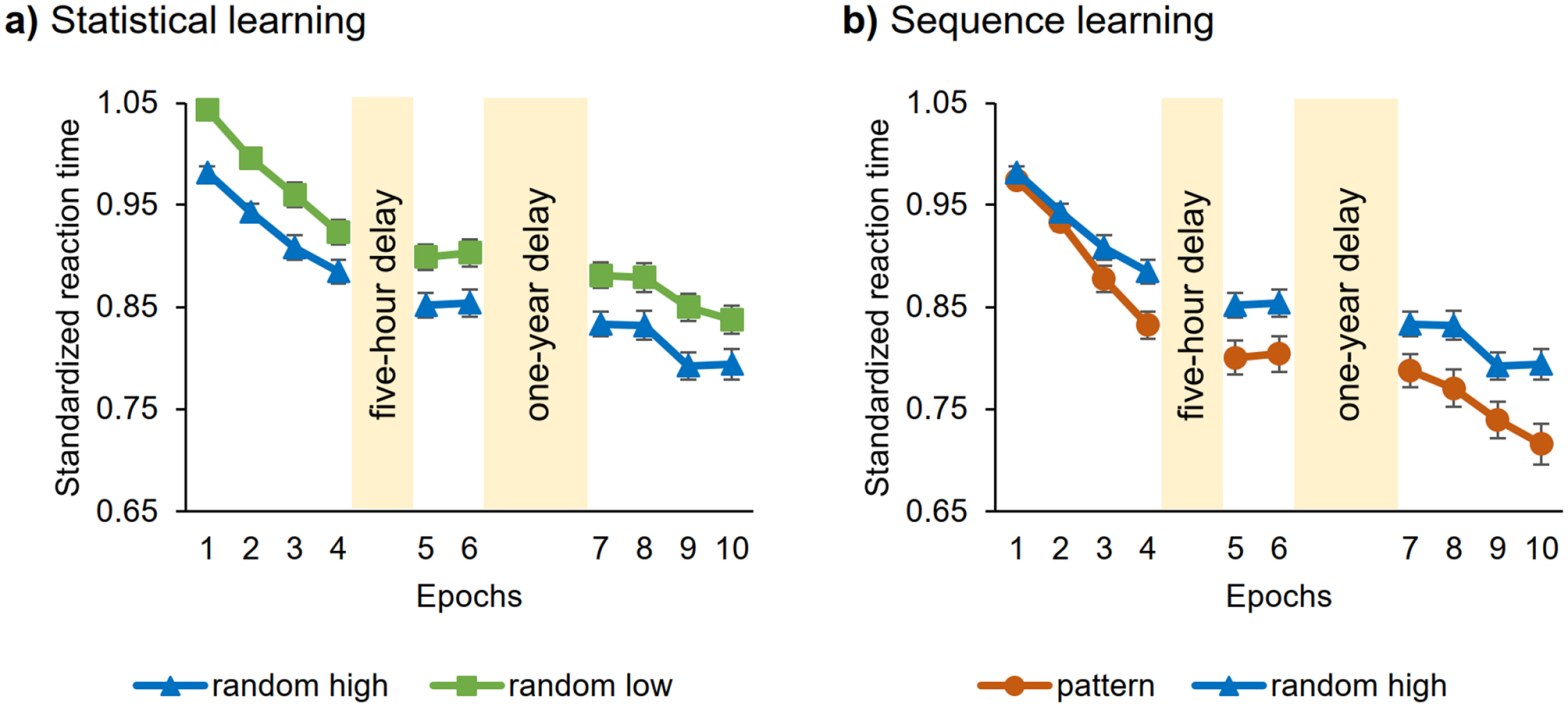
Temporal dynamics of (**a**) statistical and (**b**) sequence learning across epochs and sessions. Standardized RT values as a function of the epoch (1–10) and trial types (random high vs random low for statistical learning and pattern vs random high for sequence learning) are presented. Blue lines with triangle symbols indicate standardized RT values on the random high trials, green lines with square symbols indicate standardized RT values on the random low trials and orange lines with circle symbols indicate standardized RT values on the pattern trials. (**a**) Statistical learning is quantified by the gap between blue and green lines and (**b**) sequence learning is quantified by the gap between orange and blue lines. In both cases, greater gap between the lines represents better learning. Error bars denote standard error of mean.

**Statistical learning** during the Learning Phase was tested with a two-way repeated-measures ANOVA on RT with PROBABILITY (random high vs random low) and EPOCH (1–4) as within-subject factors. The ANOVA showed significant statistical learning (main effect of PROBABILITY, *F*(1, 69) = 128.65, *p* < 0.001, *η*^2^_*p*_ = 0.65; Fig. 2a), participants showed faster responses to random high (*M* = 0.93) compared to random low trials (*M* = 0.98). RTs gradually decreased as the task progressed, irrespective of trial types (main effect of EPOCH, *F*(3, 207) = 50.29, *p* < 0.001, *η*^2^_*p*_ = 0.42). The RT difference between random high and random low trials did not change throughout the task (non-significant PROBABILITY × EPOCH interaction, *F*(3, 207) = 2.25, *p* = 0.084).

To test **sequence learning** during the Learning Phase, a similar two-way repeated-measures ANOVA on RT with ORDER (pattern vs random high) and EPOCH (1–4) as within-subject factors were conducted. The ANOVA confirmed significant sequence learning (main effect of ORDER, *F*(1, 69) = 6.09, *p* = 0.02, *η*^2^_*p*_ = 0.08; Fig. 2b). Pairwise comparisons showed faster RTs to pattern (*M* = 0.90) than to random high trials (*M* = 0.93). RTs gradually decreased as the task progressed, irrespective of trial types (main effect of EPOCH, *F*(3, 207) = 86.85, *p* < 0.001, *η*^2^_*p*_ = 0.56). Moreover, participants were increasingly faster on pattern trials than on random high trials as the task progressed (revealed by the significant ORDER × EPOCH interaction, *F*(3, 207) = 4.43, *p* = 0.02, *η*^2^_*p*_ = 0.06).

Furthermore, to investigate whether individual differences influence the learning on the task, we correlated statistical and sequence learning scores with working memory capacity, with percentage of perseverative errors on the WCST task, with socioeconomic status, and with total problem score on the SDQ. To control for multiple comparisons, we employed False Discovery Rate correction. None of the correlations were significant (all *p*s > 0.064). We also rerun the ANOVAs on the sample without left-handed participants to control for handedness. The results were identical to the ones on the whole sample.

### Do children retain regularities after a 1-year offline period?

**Figure 3:**
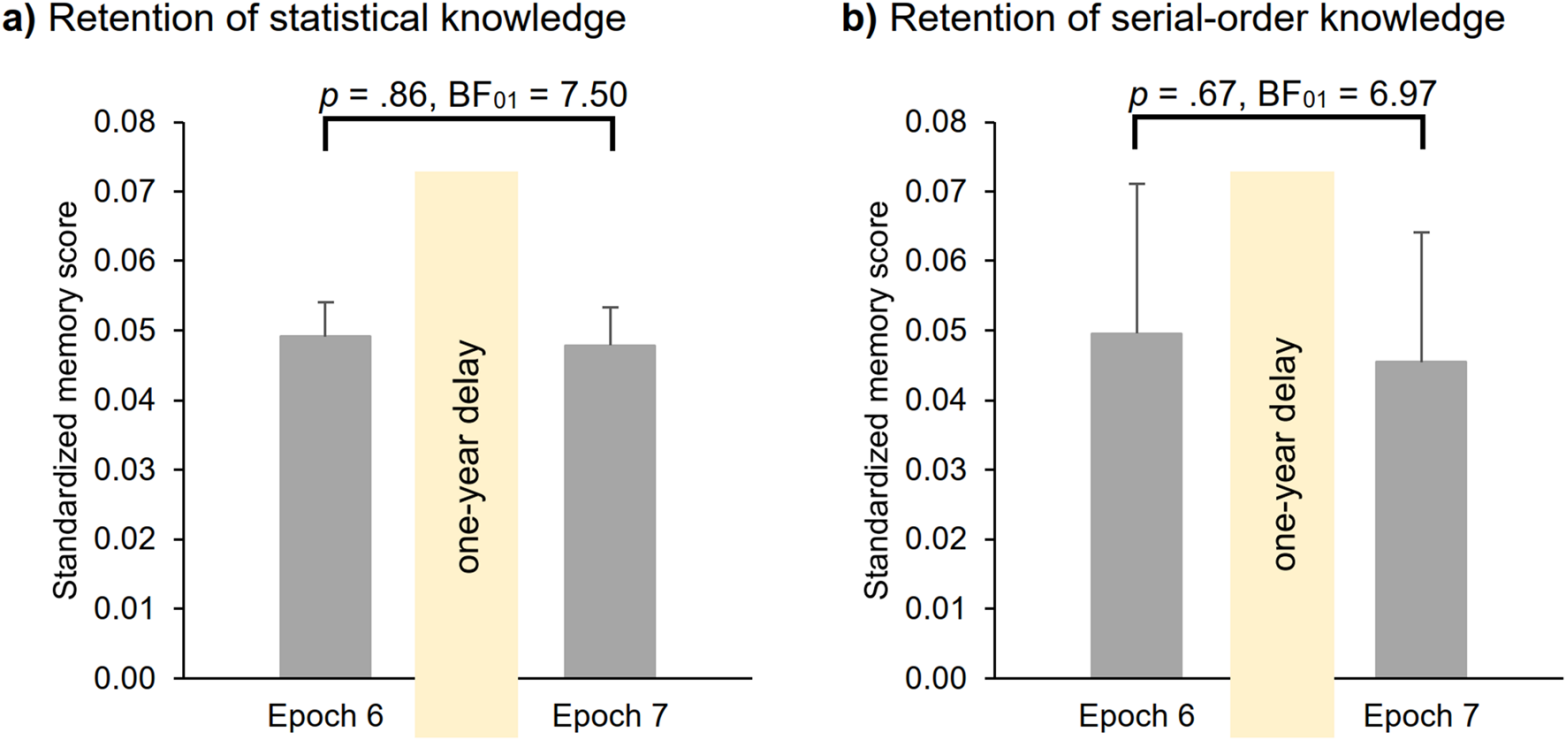
Retention of (**a**) statistical and (**b**) serial-order knowledge. Memory scores measured by standardized RT values for the last epoch of the Testing Phase (Epoch 6) and the first epoch of the Retesting Phase (Epoch 7). Error bars denote the standard error of mean.

To test 1-year consolidation of **statistical knowledge**, we conducted a two-way repeated-measures ANOVA on RT with PROBABILITY (random high vs random low) and EPOCH (6 vs. 7) as within-subject factors. Overall, irrespective of epochs, participants were faster on random high (*M* = 0.84) than on random low trials (*M* = 0.89) (main effect of PROBABILITY, *F*(1, 69) = 159.11, *p* < 0.001, *η*^2^_*p*_ = 0.70). Average RTs (i.e., RTs irrespective of trial types) differed in the two epochs (main effect of EPOCH, *F*(1, 69) = 3.92, *p* = 0.05, *η*^2^_*p*_ = 0.05), participants showed faster RTs in the 7^th^ epoch (*M* = 0.86) compared to the 6^th^ epoch (*M* = 0.88). Crucially, the ANOVA revealed evidence for retained statistical memory after the 1-year delay (non-significant PROBABILITY × EPOCH interaction, *F*(1, 69) = 0.03, *p* = 0.86, BF_01_ = 7.50; Fig. 3a), with similar memory scores in the 6^th^ (*M* = 0.049) and in the 7^th^ (*M* = 0.048) epochs. Furthermore, as the delay has some variability in terms of weeks (*M*_delay_ = 53.08 weeks, *SD*_delay_ = 2.39 weeks, between 47.95 and 60.24 weeks), we examined whether it has any relation to the long-term memory performance. First, we calculated an offline change score for statistical knowledge by subtracting the standardized memory score in Epoch 6 from the standardized memory score in Epoch 7. This way, negative scores indicate forgetting and positive scores indicate offline learning. Offline change score did not show correlation with the length of the long-term delay (*rs*(68) = 0.07, *p* = 0.56; BF_01_ = 5.12).

To investigate 1-year consolidation of **serial-order knowledge**, we also run a two-way repeated-measures ANOVA on RT with ORDER (pattern vs random high) and EPOCH (6 vs. 7) as within-subject factors. Overall, participants showed faster RTs on pattern (*M* = 0.80) than on random high trials (*M* = 0.84) (main effect of ORDER, *F*(1, 69) = 5.88, *p* = 0.02, *η*^2^_*p*_ = *0.0*8). Average RTs were similar in the two epochs (main effect of EPOCH, *F*(1, 69) = 3.33, *p* = 0.07). Importantly, the ANOVA revealed retained serial-order knowledge (non-significant ORDER × EPOCH interaction, *F*(1, 69) = 0.18, *p* = 0.67, BF_01_ = 6.97; Fig. 3b), memory scores were similar in the 6^th^ (*M* = 0.05) and in the 7^th^ epoch (*M* = 0.045). Similarly to statistical knowledge, we also correlated the offline change score of serial-order knowledge and the length of the long-term delay. One participant had to be excluded from the analysis as they showed extremely low offline change score according to Tukey^39^ criterion (more than 1.5 times the interquartile range from the quartiles). Offline change scores did not correlate with the length of the delay (*rs*(67) = −0.09, *p* = 0.49; BF_01_ = 5.01).

Moreover, similarly for the learning scores, to investigate whether individual differences influence the consolidation of statistical or serial-order knowledge, we correlated the offline change scores with working memory capacity, with percentage of perseverative errors on the WCST task, with socioeconomic status, and with total problem score on the SDQ. To control for multiple comparisons, we employed False Discovery Rate correction. None of the correlations reached significance (all *p*s > 0.277). We also rerun the ANOVAs on the sample without left-handed participants to control for handedness. The results were identical to the ones on the whole sample.

### Does age affect the one-year retention of statistical and serial-order regularities?

To check the possible association between age and retention, we conducted Pearson’s correlation between the offline change scores and age. Regarding statistical knowledge, offline change scores did not show correlation with age (*r*(68) = 0.06, *p* = 0.62, BF_01_ = 5.92). Concerning serial-order knowledge, one participant had to be excluded from the analysis on RT data as they showed extremely low offline change score according to Tukey^39^ criterion (more than 3 times the interquartile range from the quartiles). The correlation between the offline change score of serial-order knowledge represented by RT values and age was also not significant (*r*(67) = −0.06, *p* = 0.62, BF_01_ = 5.91).

## Discussion

The present study aimed to investigate the 1-year consolidation of probability-based and serial order-based regularities in children aged 9–15 years. We have shown retained knowledge of both information after the 1-year offline period; participants successfully learnt and stabilized the regularities, and the acquired knowledge was resistant to forgetting over a long period of time. Additionally, successful retention was irrespective of age as we have not found association between the retention of probability-based or serial order-based regularities and age. Our results are supported by Bayesian statistics as well, further strengthening the evidence for successful 1-year retention.

Both statistical and serial-order knowledge has been successfully retained after the 1-year offline period, which is in accordance with previous adult studies^26,27^. However, Romano et al.^26^ and Kóbor et al.^27^ employed the original, uncued version of the ASRT task in which probability-based and serial order-based regularities are intermixed. Here, we went beyond these studies by employing the cued version of the ASRT task which is designed to disentangle these two types of regularities. This version dissects probability-based and serial order-based regularities by marking pattern and random elements with different visual stimuli. This modification results in the possibility of measuring the acquisition of probability-based and serial order-based regularities (i.e., statistical and sequence learning, respectively) within the same experimental design and within the same time window^21^. It is important to note that the dissection of probability-based and serial order-based regularities in the ASRT task is possible even in the original, uncued version of the task (i.e., where pattern and random stimuli are not visually distinguishable), however, excessive training (i.e., 4-day-long practice) is needed to reach that aim^38,47^. By implementing the cued version of the ASRT task in our study design, we could simultaneously measure the encoding and consolidation of probability-based and serial order-based information, enabling us to compare the consolidation of these two processes. Although several empirical studies have shown the differences between statistical and sequence learning in encoding, to the best of our knowledge, only two studies have investigated the consolidation of these processes within the same experimental design. Simor et al.^17^ have found retained knowledge after a 1-h offline period with no difference on the behavioral level between statistical and serial-order knowledge. However, on the neural level, they discovered that slow frequency oscillations (high delta and theta power) during sleep predicted further improvements in sequence learning, while changes in statistical learning were not associated with spectral EEG power measures. Zavecz et al.^25^ also explored the brain activity underlying the consolidation of probability-based and serial order-based information. Although the consolidation of probability-based and serial order-based information was comparable on the behavioral level showing successful retention of both types of knowledge, differences emerged on the neural level. Consolidation of statistical knowledge was in relation with learning-induced changes in delta frequency connectivity between local, short-range connections, while consolidation of serial-order knowledge was associated with learning-induced changes in alpha frequency connectivity over long-range centro-parietal networks. Taken together, the present study corroborates these findings as we also showed retention of statistical and serial-order knowledge after a 1-year offline period on the behavioral level. Further studies are warranted to examine brain activity underlying long-term consolidation of probability-based and serial order-based information.

The findings of retained statistical and serial-order knowledge in children after a long period of time extends previous studies showing retention or even offline learning over the short or medium term. In more detail, Fischer et al.^31^ showed retained statistical knowledge after an 11-h offline period spent awake; whereas Desmottes et al.^33^ investigated sequence-specific learning and found offline learning both after 24-h and 1-week delay, and Hedenius et al.^32^ found retained serial-order knowledge following a 24-h delay. Four studies^34–37^ employed the uncued ASRT task (intermixing probability-based and serial order-based regularities). They found retained knowledge following a 16-h^35,37^ and 3-day^34^ offline period, and offline learning following a 24-h delay^36^. Our results on RT data are consistent with these studies. Although here we focused on RT data, analyses on accuracy data also yielded similar results (see Supplementary Material). Altogether, our results corroborate and extend the previous ones with showing successful retention after a 1-year long offline period.

Although unveiling lifespan differences in the consolidation of statistical and serial-order knowledge was not the goal of the present study, it is worth noting that our results are in line with the findings of Romano et al.^26^ and Kóbor et al.^27^, showing successful 1-year retention in adults. The development and lifespan trajectory of the *acquisition* of probability-based and serial order-based information underwent thorough investigation [e.g.,^21,29,55,56^]; however, no consensus emerged whether learning is age-dependent or not [for a review, see^50^]. Nemeth et al.^21^ examined the acquisition of probability-based and serial order-based regularities employing both the uncued and the cued ASRT task in a population of neurotypicals between the age of 11 and 39. In the uncued condition, 11–13-year-old children showed higher extent of statistical learning compared to the other age groups. This falls in line with the ‘less is more’ model, which proposes age-dependent learning of regularities with a peak performance during childhood, up until the age of 12^29,30^. In contrast, statistical learning was age-invariant in the cued condition. Sequence learning was similar in all age groups in both the cued and uncued conditions^21^, suggesting that the acquisition of serial order-based information is comparable from childhood to adulthood. Here, we went beyond the study of Nemeth et al.^21^ by investigating the *consolidation* of probability-based and serial order-based regularities. Importantly, not only learning but consolidation of statistical and serial-order knowledge could also differ during the lifespan [e.g.,^31,57^]. Fischer et al.^31^ showed age-dependent consolidation of statistical knowledge in the case of sleep-dependent consolidation. Adults benefited from sleep and showed better consolidation of statistical knowledge after sleep than wakefulness, while the exact opposite picture emerged in children [however, for the confounding effect of pre-sleep level performance, see^58^]. As for the consolidation of serial-order knowledge, the results of Adi‐Japha et al.^57^ suggests a more nuanced picture: memory performance in childhood and adulthood on the behavioral level appeared similar, showing retention of knowledge in both age groups, however, children seemed to be less susceptible to subsequent interference than adults. In the present study, we found long-lasting representation of statistical and serial-order knowledge, similarly to the studies of Romano et al.^26^ and Kóbor et al.^27^ that showed retained statistical knowledge following a 1-year offline period in adults. Thus, our study offers indirect evidence of comparable consolidation of probability-based and serial order-based information in childhood and adulthood, supporting developmental invariance in consolidation. The lack of association between retention and age in our sample also promotes the developmental invariance model. Nevertheless, as we did not directly contrast the performance of adult and children populations, further studies are warranted to examine the long-term memory performance of statistical and serial-order knowledge in adults and children within the same experimental design.

It is also worth looking at our results from a broader perspective of memory consolidation, namely the distinction between procedural and declarative processes. Statistical and serial-order regularities have been proposed to be two aspects of procedural memory, i.e., the system underlying the acquisition of skills and habits^21,38^. Hence, our results, together with previous studies on adults, suggest that consolidation of procedural knowledge is age-invariant and comparable from childhood to adulthood, both after short-term and long-term delay. The developmental differences of declarative memory, i.e., the system underlying the learning and remembering of facts and events, has also been investigated across the lifespan. While declarative memory abilities have been extensively shown to improve across childhood and adolescence, particularly memory for contextual details [e.g.,^59–61^], the developmental differences of declarative memory consolidation using relatively long offline periods (i.e., more than 24 h) have been tested only in a handful of studies. For example, Henderson et al.^62^ showed that children have retained knowledge of objects’ locations following a 1-week delay. Relatedly, in school-aged children, cued recall of previously learnt novel words was maintained^63^ or even improved^62^ after a 1-week offline period. Recognition of novel words was also maintained after a 1-week delay^62^. Similarly, in neurotypical adults, Gaskell and Dumay^64^ showed retained explicit recognition of priorly acquired novel words and Dumay et al.^65^ found increased free recall of novel words after a 1-week offline period. In sum, similarly to procedural memory, long-term consolidation of declarative memory (1-week delay in these examples) seems to be comparable between school-aged children and adults, at least considering the learning and recalling of novel words. Note that, to the best of our knowledge, no 1-year consolidation has been tested for this (or other) aspect of declarative memory in children that would allow greater comparability. Future studies are warranted to directly compare the developmental trajectory of the long-term consolidation of procedural and declarative memory within the same groups using a range of offline delays.

In the present study, we took into consideration several possible confounds of consolidation. Participants completed the task three times: (1) in the Learning Phase, (2) in the Testing Phase 5 h later on the same day and (3) in the Retesting Phase 1 year later, with no practice during the offline periods. By employing this study design, we controlled for the following possible confounds. First, by implementing a Testing Phase in the design, we controlled for the short-term (5-h) consolidation of information. Second, as participants were unaware of the fact that they will be tested with the same task later, any confounding effects of explicit strategy during acquisition or consolidation were minimized. Lastly, during the offline periods, there was no practice, which could have led to the reactivation of the acquired knowledge. Moreover, we took into account the possible confounding effect of individual differences on consolidation. We correlated consolidation performance with working memory capacity, executive functions, socioeconomic status, behavioral and emotional problems, and examined the role of handedness as well. We did not find any relation between these factors and consolidation performance; therefore, it is highly unlikely that individual differences confounded our results.

Taken together, the present study demonstrated that the representation of statistical and serial order-based regularities remains stable over a long period of time in neurotypical children and can be successfully retained after a 1-year offline period. We showed that the knowledge of statistical and serial order-based regularities is robust and resistant to forgetting over a 1-year offline period, with no difference between the two aspects of learning. Our study also offers indirect evidence for the developmental invariance of consolidation of statistical and serial-order knowledge.

## Supplementary Information

Supplementary Information.
