# Supplementary material for: Statistical and sequence learning lead to persistent memory in children after a one-year offline period
Source: Sci Rep. 2021 Jun 14;11:12418. doi: 10.1038/s41598-021-90560-5 (PMC8203620; doi:10.1038/s41598-021-90560-5)
Supplement: Supplementary file 1 — Supplementary Information. [file 41598_2021_90560_MOESM1_ESM.pdf]

Supplementary Materials

Statistical and sequence learning lead to persistent memory in children after a one-year offline period

Eszter Tóth-Fáber[a,b,c], Karolina Janacsek[b,c,d +], Dezső Németh[b,c,e +,]
[+]Shared senior authorship

Analysis of accuracy data

In the ASRT task, participants were provided with feedback about their performance, i.e., about their average RTs and accuracy, after each block. They were encouraged to keep accuracy above 92%, and the mean accuracy in the study was 92.29 % (*SD* = 3.38 %). High accuracy scores and relatively low variability in samples of neurotypical participants can hinder the detection of learning[1]; therefore, RTs could be considered a more appropriate measure of statistical and sequence learning. Based on this argument, we reported only the RT data in the Manuscript. Here, we report the analyses on accuracy values, which revealed similar results as the results on RT data.

Statistical analysis

Similarly to RT values (see Statistical analyses section of the Manuscript), prior developmental studies showed that age has a large effect on average accuracy[2-4]. To test this, we first calculated average accuracy over the 10 epochs (i.e., accuracy data was calculated on all trials, irrespective of trial types). We then correlated the average accuracy with age, which revealed a significant positive correlation ($r(68) = .32$, $p = .007$), showing that younger children were less accurate on the task. To control for the effect of average accuracy differences related to age on learning and consolidation of knowledge, we transformed the data in the following way. We divided each participants' raw accuracy values of each trial type and each epoch by

their own average performance (i.e., average accuracy) in the first epoch of the task (for a similar approach, see[5,6]). Participants' performance was around 1 at the beginning of the task and changed as the task progressed. Values above 1 indicated that responses were more accurate on a given trial type than the responses combined to all trial types (i.e., average accuracy) in the very first epoch of the task; and values below 1 meant that responses were less accurate on a given trial type compared to average accuracy in the first epoch. We conducted all analyses in the Supplementary Material on standardized accuracy.

Statistical learning score in the Learning Phase and memory scores in the Testing and Retesting Phases were quantified as the difference between random high and random low trial types in accuracy (accuracy for random high minus accuracy for random low trials). The learning and memory scores of sequence learning were calculated as the difference between pattern and random high trial types in accuracy (accuracy for pattern minus accuracy for random high trials). Higher scores indicate larger statistical or sequence learning/memory. To assess learning and the retention of knowledge, repeated measures ANOVAs and paired-samples t-tests were conducted on standardized accuracy data, separately for statistical and sequence learning. The Greenhouse-Geisser epsilon (ε) correction was used when necessary. Original *df* values and corrected *p* values (if applicable) are reported with partial eta-squared (η^2_p) as a measure of effect size. In conjunction with the frequentist analyses, we performed Bayesian paired-samples t-tests and calculated the Bayes Factor (BF) for the relevant comparisons as well.

Results

Prerequisite of memory consolidation

To assess memory consolidation, significant learning has to occur preceding the offline period. Therefore, as a first step, we conducted repeated-measures ANOVAs on the Learning

Phase to confirm that significant learning has occurred concerning both statistical and sequence learning. ANOVAs were conducted on standardized accuracy separately for statistical and sequence learning.

Statistical learning during the Learning Phase wase tested with a two-way repeated-measures ANOVA with PROBABILITY (random high vs random low) and EPOCH (1-4) as within-subject factors. The ANOVA revealed significant statistical learning (main effect of PROBABILITY, $F(1, 69) = 33.65$, $p < .001$, $\eta^2_p = .33$). Post-hoc pairwise comparisons revealed higher accuracy on random high trials ($M = 1.003$) compared to random low trials ($M = 0.97$). Average accuracy (i.e., irrespective of trial types) did not change throughout the task (main effect of EPOCH, $F(3, 207) = 1.54$, $p = .22$). Statistical learning also did not change as the task progressed (PROBABILITY × EPOCH interaction, $F(3, 207) = 0.93$, $p = .43$, Fig. S1A).

To test **sequence learning** during the Learning Phase, similar two-way repeated-measures ANOVAs with ORDER (pattern vs random high) and EPOCH (1-4) as within-subject factors were conducted. The ANOVA revealed marginally significant learning (main effect of ORDER, $F(1, 69) = 3.39$, $p = .07$, $\eta^2_p = .05$), participants showed marginally higher accuracy on pattern ($M = 1.01$) compared to random high trials ($M = 1.003$). Neither the average accuracy, nor the extent of sequence learning changed throughout the task (main effect of EPOCH, $F(3, 207) = 1.46$, $p = .24$ and ORDER × EPOCH interaction, $F(3, 207) = 2.39$, $p = .07$, respectively, Fig. S1B).

Furthermore, to investigate whether individual differences influence the learning on the task, we correlated statistical and sequence learning scores with working memory capacity, with percentage of perseverative errors on the WCST task, with socioeconomic status, and with total problem score on the SDQ. To control for multiple comparisons, we employed False Discovery Rate correction. None of the correlations reached significance (all *ps* > .128). We also rerun the

ANOVAs on the sample without left-handed participants to control for handedness. The results were identical to the ones on the whole sample.

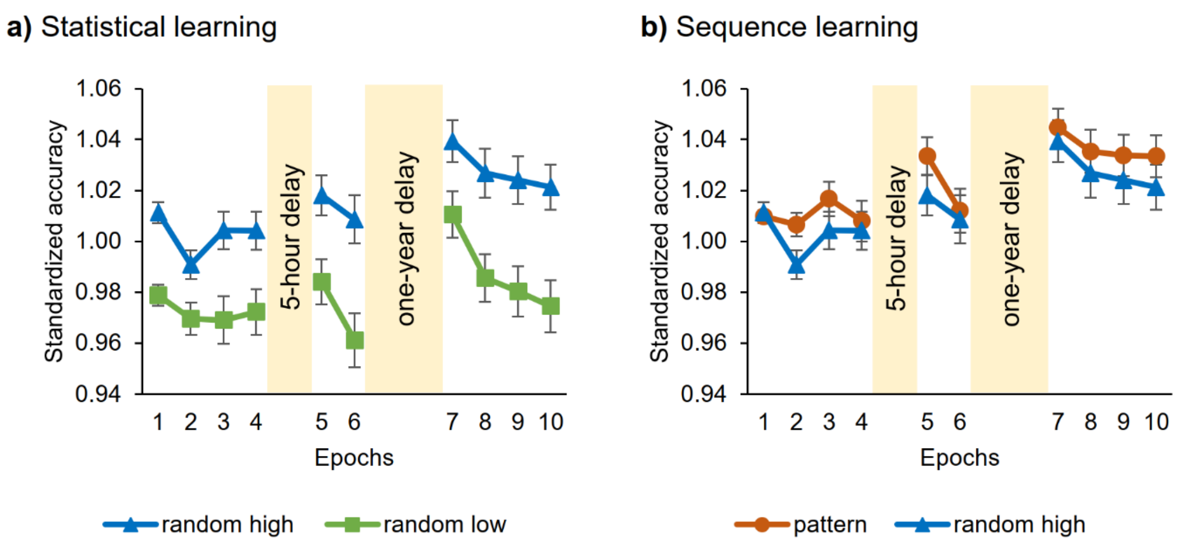

Figure S1. Temporal dynamics of (A) statistical and (B) sequence learning across epochs and sessions. Standardized accuracy values as a function of the epoch (1-10) and trial types (random high vs random low for statistical learning and pattern vs random high for sequence learning) are presented. Blue lines with triangle symbols indicate standardized accuracy values on the random high trials, green lines with square symbols indicate standardized accuracy values on the random low trials and orange lines with circle symbols indicate standardized accuracy values on the pattern trials. (A) Statistical learning is quantified by the gap between blue and green lines and (B) sequence learning is quantified by the gap between orange and blue lines. In both cases, greater gap between the lines represents better learning. Error bars denote standard error of mean.

Do children retain regularities after a one-year offline period?

To test one-year retention of **statistical knowledge**, we conducted a two-way repeated-measures ANOVA with PROBABILITY (random high vs random low) and EPOCH (6 vs. 7) as within-subject factors. Overall, irrespective of epochs, participants showed higher accuracy on random high (M = 1.02) compared to random low trials (M = 0.99) (main effect of PROBABILITY, $F(1, 69) = 49.53$, $p < .001$, $\eta^2_p = .42$). Average accuracy (i.e., irrespective of trial types) differed in the two epochs (main effect of EPOCH, $F(1, 69) = 30.10$, $p < .001$, η^2_p

= .30), participants showed higher accuracy in the 7th epoch (M = 1.03) than in the 6th epoch (M = 0.99). The ANOVA showed a difference in memory scores between the Testing and Retesting Phases (significant PROBABILITY × EPOCH interaction, $F(1, 69) = 4.34$, $p = .04$, $\eta^2_p = .06$) Follow-up paired sample t-tests on the memory scores revealed that statistical memory underwent decrease over the one-year delay (6th epoch: M = 0.0474, 7th epoch: M = 0.0288; Fig. S2A), however, Bayesian analysis did not confirm this result (BF_{01} = 1.003). Furthermore, as the long-term delay had some variability in terms of weeks (M_{delay} = 53.08 weeks, SD_{delay} = 2.39 weeks, between 47.95 and 60.24 weeks), we examined whether it has any relation to the long-term memory performance. First, we calculated an offline change score for statistical knowledge by subtracting the standardized memory score in Epoch 6 from the standardized memory score in Epoch 7. This way, negative scores indicate forgetting, and positive scores indicate offline learning. Offline change score did not show correlation with the length of the long-term delay ($rs(68) = .129$, $p = .287$; BF_{01} = 4.023).

To investigate one-year retention of **serial-order knowledge**, we also ran a two-way repeated-measures ANOVA with ORDER (pattern vs random high) and EPOCH (6 vs. 7) as within-subject factors. Overall, irrespective of epoch, participants showed comparable accuracy on pattern and random high trials (main effect of PROBABILITY, $F(1, 69) = 1.71$, $p = .20$). Average accuracy (i.e., irrespective of trial types) differed in the two epochs (main effect of EPOCH, $F(1, 69) = 29.48$, $p < .001$, $\eta^2_p = .30$), participants showed higher accuracy in the 7th epoch (M = 1.04) than in the 6th epoch (M = 1.01). The ANOVA revealed evidence for persistent memory representations of serial-order knowledge (non-significant ORDER × EPOCH interaction, $F(1, 69) = 0.06$, $p = .81$, BF_{01} = 7.404). Follow-up paired sample t-tests on the memory scores showed comparable serial-order knowledge in the Testing and Retesting Phases (6th epoch: M = 0.0035, 7th epoch: M = 0.0054; Fig. S2B). Similarly to statistical knowledge, we also correlated the offline change score of serial-order knowledge and the length of the long-

term delay. Offline change scores of serial-order knowledge did not correlate with the length of the delay ($r_s(68) = -.190$, $p = .114$; $BF_{01} = 2.049$).

Moreover, similarly for the learning scores, to investigate whether individual differences influence the consolidation of statistical or serial-order knowledge, we correlated the offline change scores with working memory capacity, with percentage of perseverative errors on the WCST task, with socioeconomic status and with total problem score on the SDQ. To control for multiple comparisons, we employed False Discovery Rate correction. None of the correlations reached significance (all *ps* > .766). We also rerun the ANOVAs on the sample without left-handed participants to control for handedness. The results were identical to the ones on the whole sample.

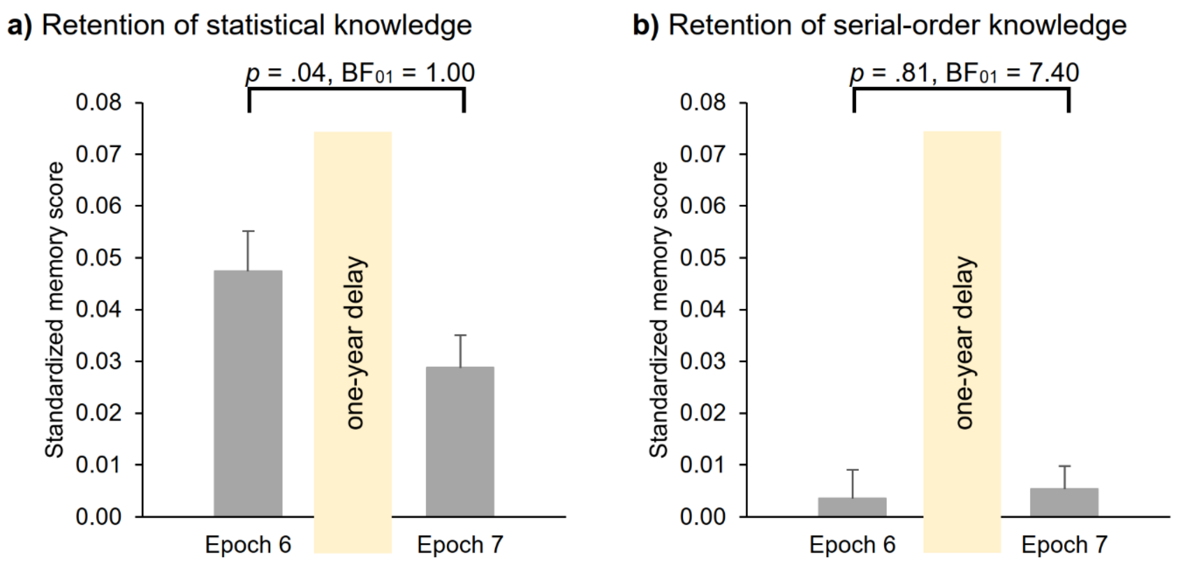

Figure S2. Retention of (A) statistical and (B) serial-order knowledge. Memory scores measured by standardized accuracy values for the last epoch of the Testing Phase (Epoch 6) and the first epoch of the Retesting Phase (Epoch 7). Error bars denote the standard error of mean.

Does age affect the one-year retention of statistical and serial-order regularities?

To check the possible association between age and retention, we conducted Pearson's correlation between the offline change scores and age. Regarding statistical knowledge, offline change scores did not show correlation with age ($r(68) = .01$, $p = .92$, $BF_{01} = 6.67$). Concerning serial-order knowledge, offline change scores in accuracy also did not correlate with age ($r(68) = .15$, $p = .21$, $BF_{01} = 3.08$).

References

1. Vékony, T. *et al.* Speed or accuracy instructions during skill learning do not affect the acquired knowledge. *Cerebral Cortex Communications* **1**, tgaa041 (2020).
2. Janacsek, K., Fiser, J. & Nemeth, D. The best time to acquire new skills: age-related differences in implicit sequence learning across the human lifespan. *Dev. Sci.* **15**, 496-505 (2012).
3. Zwart, F. S., Vissers, C. T. W., Kessels, R. P. & Maes, J. H. Procedural learning across the lifespan: A systematic review with implications for atypical development. *J. Neuropsychol.* **13**, 149-182 (2019).
4. Juhasz, D., Nemeth, D. & Janacsek, K. Is there more room to improve? The lifespan trajectory of procedural learning and its relationship to the between- and within-group differences in average response times. *PLoS One* **14**, e0215116, doi:10.1371/journal.pone.0215116 (2019).
5. Nitsche, M. A. *et al.* Facilitation of implicit motor learning by weak transcranial direct current stimulation of the primary motor cortex in the human. *J. Cogn. Neurosci.* **15**, 619-626 (2003).
6. Horvath, K., Torok, C., Pesthy, O., Nemeth, D. & Janacsek, K. Divided attention does not affect the acquisition and consolidation of transitional probabilities. *Sci. Rep.*, doi:10.1038/s41598-020-79232-yda129685-49a7-438c-85e0-20e6384f29e9 (2021).
